# 4-Meth­oxy­benzamidinium acetate

**DOI:** 10.1107/S1600536812044911

**Published:** 2012-11-03

**Authors:** Simona Irrera, Gustavo Portalone

**Affiliations:** a Chemistry Department, "Sapienza" University of Rome, P.le A. Moro, 5, I-00185 Rome, Italy

## Abstract

The title compound, C_8_H_11_N_2_O^+^·CH_3_CO_2_^−^, was synthesized by a reaction between 4-meth­oxy­benzamidine (4-amidino­anisole) and acetic acid. In the cation, the amidinium group forms a dihedral angle of 11.65 (17)° with the mean plane of the benzene ring. The ionic components are associated in the crystal *via* N—H^+^⋯O^−^ hydrogen bonds, resulting in a one-dimensional structure consisting of dimers and catemers and orientated approximately along the *c* axis.

## Related literature

For the biological and pharmacological relevance of benzamidine, see: Powers & Harper (1999 ▶). For structural analysis of proton-transfer adducts containing mol­ecules of biological inter­est, see: Portalone, (2011*a* ▶); Portalone & Irrera (2011 ▶). For the supra­molecular association in proton-transfer adducts containing benzamidinium cations, see; Portalone (2010 ▶, 2011*b* ▶, 2012 ▶); Irrera & Portalone (2012*a* ▶,*b* ▶); Irrera *et al.* (2012 ▶). For hydrogen-bond motifs, see Bernstein *et al.* (1995 ▶). For standard bond lengths, see: Allen *et al.* (1987 ▶).
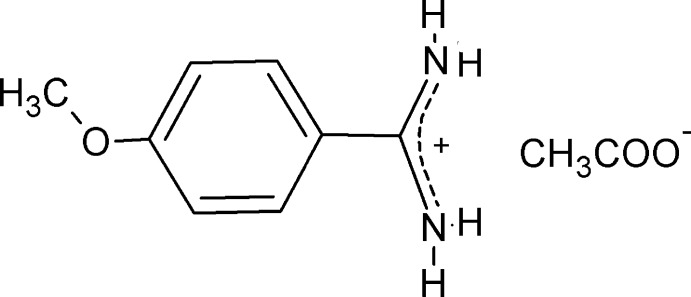


## Experimental

### 

#### Crystal data

C_8_H_11_N_2_O^+^·C_2_H_3_O_2_^−^
*M* *_r_* = 210.23
Monoclinic, 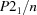

*a* = 8.7591 (14) Å
*b* = 6.5478 (8) Å
*c* = 19.456 (3) Å
β = 102.580 (14)°
*V* = 1089.0 (3) Å^3^
*Z* = 4
Mo *K*α radiation
μ = 0.10 mm^−1^
*T* = 298 K
0.21 × 0.18 × 0.15 mm

#### Data collection

Oxford Diffraction Xcalibur S CCD diffractometer
Absorption correction: multi-scan (*CrysAlis PRO*; Agilent, 2011 ▶) *T*_min_ = 0.980, *T*_max_ = 0.986
14433 measured reflections
2365 independent reflections
1834 reflections with *I* > 2σ(*I*)
*R*_int_ = 0.040

#### Refinement

*R*[*F* ^2^ > 2σ(*F* ^2^)] = 0.056
*wR*(*F* ^2^) = 0.141
*S* = 1.08
2365 reflections
156 parameters
H atoms treated by a mixture of independent and constrained refinement
Δρ_max_ = 0.21 e Å^−3^
Δρ_min_ = −0.15 e Å^−3^

### 

Data collection: *CrysAlis PRO* (Agilent, 2011 ▶); cell refinement: *CrysAlis PRO*; data reduction: *CrysAlis PRO*; program(s) used to solve structure: *SIR97* (Altomare *et al.*, 1999 ▶); program(s) used to refine structure: *SHELXL97* (Sheldrick, 2008 ▶); molecular graphics: *ORTEP-3* (Farrugia, 1997 ▶); software used to prepare material for publication: *WinGX* (Farrugia, 1999 ▶).

## Supplementary Material

Crystal structure: contains datablock(s) global, I. DOI: 10.1107/S1600536812044911/tk5166sup1.cif

Structure factors: contains datablock(s) I. DOI: 10.1107/S1600536812044911/tk5166Isup2.hkl

Additional supplementary materials:  crystallographic information; 3D view; checkCIF report

## Figures and Tables

**Table 1:** Hydrogen-bond geometry (Å, °)

*D*—H⋯*A*	*D*—H	H⋯*A*	*D*⋯*A*	*D*—H⋯*A*
N1—H1*A*⋯O1	0.91 (3)	1.94 (3)	2.847 (2)	175 (2)
N1—H1*B*⋯O1^i^	0.91 (2)	1.98 (2)	2.832 (2)	155 (2)
N2—H2*A*⋯O2	0.94 (3)	1.83 (3)	2.776 (2)	176 (2)
N2—H2*B*⋯O2^ii^	0.88 (2)	1.95 (2)	2.817 (2)	168 (2)

Symmetry codes: (i) 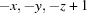; (ii) 
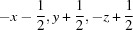
.

**Fig. 1.**
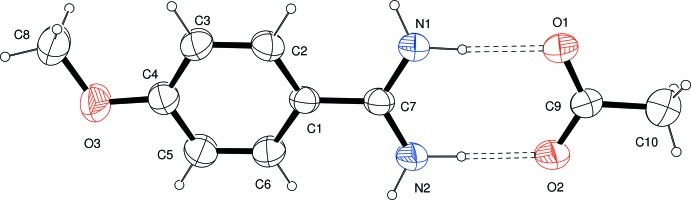
The asymmetric unit of (I), showing the atom-labelling scheme. Displacements ellipsoids are at the 50% probability level. The asymmetric unit was selected so that the two ions are linked by N+.—H···O- hydrogen bonds. H atoms are shown as small spheres of arbitrary radii. Hydrogen bonding is indicated by dashed lines.

**Fig. 2.**
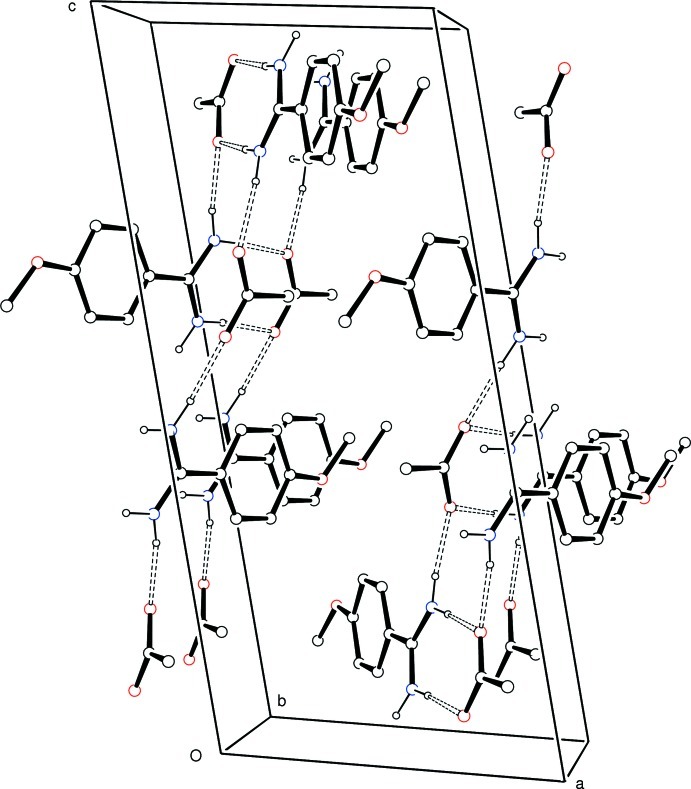
Crystal packing diagram for (I), viewed approximately down b. Displacements ellipsoids are at the 50% probability level. H atoms are shown as small spheres of arbitrary radii. For the sake of clarity, H atoms not involved in hydrogen bonding have been omitted. Hydrogen bonding is indicated by dashed lines.

## supplementary crystallographic information

### Comment

The present work is part of a general structural analysis of proton-transfer adducts containing molecules of biological interest (Portalone, 2011*a*; Portalone & Irrera, 2011). In this context, benzamidine derivatives, which have shown strong biological and pharmacological activity (Powers & Harper, 1999), are being used in our group as bricks for supramolecular construction (Portalone, 2010, 2011*b*, 2012). Indeed, the bidentate hydrogen-bonding interaction between the amidinium and the carboxylate functional groups can be a powerful organizing force in solution and in the solid-state.

We report here the single-crystal structure of the title molecular salt, 4-methoxybenzamidinium acetate, (I), which was obtained by a reaction between 4-methoxybenzamidine (4-amidinoanisole) and acetic acid.

The asymmetric unit of (I) comprises one non-planar 4-methoxybenzamidinium cation and one acetate anion (Fig. 1).

In the cation the amidinium group forms dihedral angle of 11.65 (17)° with the mean plane of the phenyl ring, which agrees with the values observed in protonated benzamidinium ions (14.4 (1) - 32.7 (1)°, Portalone, 2010, 2012; Irrera *et al.*, 2012). The lack of planarity in all these systems is obviously caused by steric hindrances between the H atoms of the aromatic ring and the amidine moiety. This conformation is rather common in benzamidinium-containing small-molecule crystal structures, with the only exception of benzamidinium diliturate, where the benzamidinium cation is planar (Portalone, 2010). The pattern of bond lengths and bond angles of the 4-methoxybenzamidinium cation agrees with that reported in previous structural investigations (Irrera *et al.*, 2012; Portalone, 2010, 2012; Irrera & Portalone, 2012*a*, 2012*b*). In particular the amidinium group, true to one's expectations, features C—N bonds within experimental error [1.312 (2) and 1.306 (2) Å], evidencing the delocalization of the π electrons and double-bond character.

In the acetate moiety the C—O bond lengths indicate delocalization of the negative charge on both O atoms, since the C—O bond lengths [1.250 (2) and 1.249 (2) Å] are intermediate between single C*sp*^2^—O and double C*sp*^2^═O, and correlate well with values for carboxylate anions [1.247 - 1.262 Å, Allen *et al.*, 1987].

Analysis of the crystal packing of (I), (Fig. 2), shows that each amidinium unit is bound to three acetate anions by four distinct N—H^+^···O^-^ strong intermolecular hydrogen bonds (N^+^···O^-^ = 2.776 (2) - 2.847 (2) Å, Table 1) into a one-dimensional structure. The ion pairs of the asymmetric unit are joined by two N^+^—H···O^-^ (±) hydrogen bonds to form ionic dimers with graph-set motif *R*^2^_2_(8) (Bernstein *et al.*, 1995). These subunits are then joined as catemers into linear chains approximately along the crystallographic *c* axis through the remaining N^+^—H···O^-^ hydrogen bonds to adjacent anti-parallel dimers.

### Experimental

4-Methoxybenzamidine (0.1 mmol, Fluka at 96% purity) was dissolved without further purification in 8 ml of a 20% solution of acetic acid and heated under reflux for 3 h. After cooling the solution to an ambient temperature, colourless crystals suitable for single-crystal X-ray diffraction were grown by slow evaporation of the solvent after one week.

### Refinement

All H atoms were identified in difference Fourier maps, but for refinement all C-bound H atoms were placed in calculated positions, with C—H = 0.93 Å (phenyl) and 0.89 - 1.01 Å (methyl), and refined as riding on their carrier atoms. The *U*_iso_ values were kept equal to 1.2*U*_eq_(C, phenyl). and to 1.5*U*_eq_(C, methyl). Positional and displacement parameters of H atoms of the amidinium group were refined, giving N—H distances in the range 0.88 (2) - 0.94 (3) Å.

### Crystal data

**Table:**

C_8_H_11_N_2_O^+^·C_2_H_3_O_2_^−^	*F*(000) = 448
*M**_r_* = 210.23	*D*_x_ = 1.282 Mg m^−^^3^
Monoclinic, *P*2_1_/*n*	Mo *K*α radiation, λ = 0.71073 Å
Hall symbol: -P 2yn	Cell parameters from 3220 reflections
*a* = 8.7591 (14) Å	θ = 2.8–28.9°
*b* = 6.5478 (8) Å	µ = 0.10 mm^−^^1^
*c* = 19.456 (3) Å	*T* = 298 K
β = 102.580 (14)°	Tablets, colourless
*V* = 1089.0 (3) Å^3^	0.21 × 0.18 × 0.15 mm
*Z* = 4	

### Data collection

**Table:**

Oxford Diffraction Xcalibur S CCD diffractometer	2365 independent reflections
Radiation source: Enhance (Mo) X-ray Source	1834 reflections with *I* > 2σ(*I*)
Graphite monochromator	*R*_int_ = 0.040
Detector resolution: 16.0696 pixels mm^-1^	θ_max_ = 27.0°, θ_min_ = 3.3°
ω and φ scans	*h* = −11→11
Absorption correction: multi-scan (*CrysAlis PRO*; Agilent, 2011)	*k* = −8→8
*T*_min_ = 0.980, *T*_max_ = 0.986	*l* = −24→24
14433 measured reflections	

### Refinement

**Table:**

Refinement on *F*^2^	Primary atom site location: structure-invariant direct methods
Least-squares matrix: full	Secondary atom site location: difference Fourier map
*R*[*F*^2^ > 2σ(*F*^2^)] = 0.056	Hydrogen site location: inferred from neighbouring sites
*wR*(*F*^2^) = 0.141	H atoms treated by a mixture of independent and constrained refinement
*S* = 1.08	*w* = 1/[σ^2^(*F*_o_^2^) + (0.0561*P*)^2^ + 0.3698*P*] where *P* = (*F*_o_^2^ + 2*F*_c_^2^)/3
2365 reflections	(Δ/σ)_max_ < 0.001
156 parameters	Δρ_max_ = 0.21 e Å^−^^3^
0 restraints	Δρ_min_ = −0.15 e Å^−^^3^

### Special details

**Table:**

Geometry. All s.u.'s (except the s.u. in the dihedral angle between two l.s. planes) are estimated using the full covariance matrix. The cell s.u.'s are taken into account individually in the estimation of s.u.'s in distances, angles and torsion angles; correlations between s.u.'s in cell parameters are only used when they are defined by crystal symmetry. An approximate (isotropic) treatment of cell s.u.'s is used for estimating s.u.'s involving l.s. planes.
Refinement. Refinement of *F*^2^ against ALL reflections. The weighted *R*-factor *wR* and goodness of fit *S* are based on *F*^2^, conventional *R*-factors *R* are based on *F*, with *F* set to zero for negative *F*^2^. The threshold expression of *F*^2^ > 2σ(*F*^2^) is used only for calculating *R*-factors(gt) *etc*. and is not relevant to the choice of reflections for refinement. *R*-factors based on *F*^2^ are statistically about twice as large as those based on *F*, and *R*- factors based on ALL data will be even larger.

### Fractional atomic coordinates and isotropic or equivalent isotropic displacement parameters (Å^2^)

**Table:**

	*x*	*y*	*z*	*U*_iso_*/*U*_eq_	
O3	0.2977 (2)	1.0070 (2)	0.35136 (8)	0.0624 (5)	
N1	−0.0153 (2)	0.1780 (3)	0.42774 (8)	0.0453 (4)	
H1A	−0.074 (3)	0.064 (4)	0.4296 (12)	0.063 (7)*	
H1B	0.054 (3)	0.217 (4)	0.4677 (13)	0.063 (7)*	
N2	−0.1170 (2)	0.2005 (3)	0.31128 (9)	0.0468 (4)	
H2A	−0.175 (3)	0.081 (4)	0.3149 (12)	0.070 (7)*	
H2B	−0.133 (3)	0.261 (3)	0.2696 (13)	0.055 (6)*	
C1	0.0643 (2)	0.4636 (3)	0.36478 (9)	0.0343 (4)	
C2	0.1370 (2)	0.5636 (3)	0.42564 (10)	0.0449 (5)	
H2	0.1318	0.5077	0.4690	0.054*	
C3	0.2173 (2)	0.7446 (3)	0.42381 (10)	0.0472 (5)	
H3	0.2659	0.8088	0.4655	0.057*	
C4	0.2248 (2)	0.8294 (3)	0.35969 (10)	0.0429 (5)	
C5	0.1539 (3)	0.7298 (4)	0.29855 (10)	0.0584 (6)	
H5	0.1594	0.7856	0.2552	0.070*	
C6	0.0756 (3)	0.5502 (3)	0.30090 (10)	0.0510 (5)	
H6	0.0293	0.4849	0.2591	0.061*	
C7	−0.0247 (2)	0.2737 (3)	0.36775 (9)	0.0356 (4)	
C8	0.3954 (3)	1.0997 (4)	0.41088 (13)	0.0624 (6)	
H8A	0.3323 (10)	1.130 (2)	0.4474 (7)	0.094*	
H8B	0.4395 (17)	1.231 (2)	0.3962 (3)	0.094*	
H8C	0.4838 (17)	1.0040 (17)	0.4315 (6)	0.094*	
O1	−0.20216 (18)	−0.1693 (2)	0.44118 (7)	0.0548 (4)	
O2	−0.29092 (18)	−0.1444 (2)	0.32662 (7)	0.0550 (4)	
C9	−0.2818 (2)	−0.2339 (3)	0.38404 (9)	0.0390 (4)	
C10	−0.3743 (3)	−0.4258 (3)	0.38417 (12)	0.0606 (6)	
H10A	−0.3120 (10)	−0.5337 (17)	0.3841 (9)	0.091*	
H10B	−0.4519 (17)	−0.4292 (13)	0.3458 (7)	0.091*	
H10C	−0.4143 (17)	−0.4291 (12)	0.4226 (7)	0.091*	

### Atomic displacement parameters (Å^2^)

**Table:**

	*U*^11^	*U*^22^	*U*^33^	*U*^12^	*U*^13^	*U*^23^
O3	0.0827 (11)	0.0586 (9)	0.0449 (9)	−0.0302 (8)	0.0113 (8)	0.0040 (7)
N1	0.0564 (11)	0.0443 (10)	0.0298 (8)	−0.0120 (8)	−0.0020 (7)	0.0052 (7)
N2	0.0613 (11)	0.0439 (10)	0.0293 (9)	−0.0095 (8)	−0.0034 (8)	0.0040 (7)
C1	0.0389 (10)	0.0354 (9)	0.0271 (9)	0.0018 (7)	0.0039 (7)	0.0009 (7)
C2	0.0616 (13)	0.0460 (11)	0.0263 (9)	−0.0063 (9)	0.0076 (8)	0.0021 (8)
C3	0.0616 (13)	0.0474 (11)	0.0302 (10)	−0.0119 (9)	0.0049 (9)	−0.0056 (8)
C4	0.0485 (11)	0.0423 (10)	0.0379 (10)	−0.0043 (8)	0.0092 (8)	0.0012 (8)
C5	0.0798 (16)	0.0647 (14)	0.0292 (10)	−0.0221 (12)	0.0083 (10)	0.0075 (9)
C6	0.0686 (14)	0.0545 (12)	0.0270 (10)	−0.0158 (10)	0.0037 (9)	−0.0019 (8)
C7	0.0411 (10)	0.0358 (9)	0.0279 (9)	0.0021 (7)	0.0030 (7)	0.0007 (7)
C8	0.0673 (15)	0.0587 (14)	0.0587 (14)	−0.0234 (11)	0.0086 (12)	−0.0064 (11)
O1	0.0657 (10)	0.0627 (9)	0.0293 (7)	−0.0192 (7)	−0.0045 (6)	0.0027 (6)
O2	0.0742 (10)	0.0551 (9)	0.0281 (7)	−0.0154 (7)	−0.0053 (7)	0.0019 (6)
C9	0.0376 (10)	0.0436 (10)	0.0330 (10)	−0.0009 (8)	0.0014 (8)	−0.0003 (8)
C10	0.0608 (14)	0.0558 (13)	0.0603 (15)	−0.0123 (11)	0.0023 (12)	0.0026 (11)

### Geometric parameters (Å, º)

**Table:**

O3—C4	1.353 (2)	C3—H3	0.9300
O3—C8	1.418 (3)	C4—C5	1.380 (3)
N1—C7	1.312 (2)	C5—C6	1.367 (3)
N1—H1A	0.91 (3)	C5—H5	0.9300
N1—H1B	0.91 (2)	C6—H6	0.9300
N2—C7	1.306 (2)	C8—H8A	1.0093
N2—H2A	0.94 (3)	C8—H8B	1.0093
N2—H2B	0.88 (2)	C8—H8C	1.0093
C1—C2	1.381 (2)	O1—C9	1.250 (2)
C1—C6	1.389 (3)	O2—C9	1.249 (2)
C1—C7	1.475 (2)	C9—C10	1.495 (3)
C2—C3	1.383 (3)	C10—H10A	0.8930
C2—H2	0.9300	C10—H10B	0.8930
C3—C4	1.380 (3)	C10—H10C	0.8930
			
C4—O3—C8	119.11 (16)	C5—C6—C1	121.01 (18)
C7—N1—H1A	120.0 (14)	C5—C6—H6	119.5
C7—N1—H1B	121.9 (15)	C1—C6—H6	119.5
H1A—N1—H1B	118 (2)	N2—C7—N1	118.69 (18)
C7—N2—H2A	119.1 (14)	N2—C7—C1	120.84 (16)
C7—N2—H2B	123.6 (14)	N1—C7—C1	120.45 (16)
H2A—N2—H2B	117 (2)	O3—C8—H8A	109.5
C2—C1—C6	117.64 (17)	O3—C8—H8B	109.5
C2—C1—C7	120.99 (16)	H8A—C8—H8B	109.5
C6—C1—C7	121.36 (16)	O3—C8—H8C	109.5
C1—C2—C3	121.77 (17)	H8A—C8—H8C	109.5
C1—C2—H2	119.1	H8B—C8—H8C	109.5
C3—C2—H2	119.1	O2—C9—O1	123.45 (18)
C4—C3—C2	119.52 (17)	O2—C9—C10	117.82 (16)
C4—C3—H3	120.2	O1—C9—C10	118.71 (17)
C2—C3—H3	120.2	C9—C10—H10A	109.5
O3—C4—C5	116.00 (17)	C9—C10—H10B	109.5
O3—C4—C3	124.78 (17)	H10A—C10—H10B	109.5
C5—C4—C3	119.22 (18)	C9—C10—H10C	109.5
C6—C5—C4	120.83 (18)	H10A—C10—H10C	109.5
C6—C5—H5	119.6	H10B—C10—H10C	109.5
C4—C5—H5	119.6		
			
C6—C1—C2—C3	0.6 (3)	C3—C4—C5—C6	0.6 (4)
C7—C1—C2—C3	−178.24 (18)	C4—C5—C6—C1	0.5 (4)
C1—C2—C3—C4	0.4 (3)	C2—C1—C6—C5	−1.1 (3)
C8—O3—C4—C5	−169.3 (2)	C7—C1—C6—C5	177.8 (2)
C8—O3—C4—C3	10.9 (3)	C2—C1—C7—N2	167.00 (19)
C2—C3—C4—O3	178.89 (19)	C6—C1—C7—N2	−11.9 (3)
C2—C3—C4—C5	−1.0 (3)	C2—C1—C7—N1	−11.3 (3)
O3—C4—C5—C6	−179.3 (2)	C6—C1—C7—N1	169.9 (2)

### Hydrogen-bond geometry (Å, º)

**Table:**

*D*—H···*A*	*D*—H	H···*A*	*D*···*A*	*D*—H···*A*
N1—H1*A*···O1	0.91 (3)	1.94 (3)	2.847 (2)	175 (2)
N1—H1*B*···O1^i^	0.91 (2)	1.98 (2)	2.832 (2)	155 (2)
N2—H2*A*···O2	0.94 (3)	1.83 (3)	2.776 (2)	176 (2)
N2—H2*B*···O2^ii^	0.88 (2)	1.95 (2)	2.817 (2)	168 (2)

Symmetry codes: (i) −*x*, −*y*, −*z*+1; (ii) −*x*−1/2, *y*+1/2, −*z*+1/2.
